# Postpartum Bonding Disorder: Factor Structure, Validity, Reliability and a Model Comparison of the Postnatal Bonding Questionnaire in Japanese Mothers of Infants

**DOI:** 10.3390/healthcare4030050

**Published:** 2016-08-02

**Authors:** Yukiko Ohashi, Toshinori Kitamura, Kyoko Sakanashi, Tomoko Tanaka

**Affiliations:** 1 Kitamura Institute of Mental Health Tokyo, Tokyo 151-0063, Japan; kitamura@institute-of-mental-health.jp; 2 Department of Nursing, Faculty of Health Science Technology, Bunkyo Gakuin University, Tokyo 113-8668, Japan; 3 Department of Psychiatry, Graduate School of Medicine, Nagoya University, Nagoya 466-8550, Japan; 4 Graduate School of Health Sciences, Kumamoto University, Kumamoto 860-0976, Japan; sakanasi@kumamoto-u.ac.jp; 5 Aso Health Center, Kumamoto Prefecture, Kumamoto 869-2301, Japan; tanaka-t-dh@pref.kumamoto.lg.jp

**Keywords:** bonding, postnatal, mother, infant, psychometric properties

## Abstract

Negative attitudes of mothers towards their infant is conceptualized as postpartum bonding disorder, which leads to serious health problems in perinatal health care. However, its measurement still remains to be standardized. Our aim was to examine and confirm the psychometric properties of the Postnatal Bonding Questionnaire (PBQ) in Japanese mothers. We distributed a set of questionnaires to community mothers and studied 392 mothers who returned the questionnaires at 1 month after childbirth. Our model was compared with three other models derived from previous studies. In a randomly halved sample, an exploratory factor analysis yielded a three-factor structure: Anger and Restrictedness, Lack of Affection, and Rejection and Fear. This factor structure was cross-validated by a confirmatory factor analysis using the other halved sample. The three subscales showed satisfactory internal consistency. The three PBQ subscale scores were correlated with depression and psychological abuse scores. Their test–retest reliability between day 5 and 1 month after childbirth was measured by intraclass correlation coefficients between 0.76 and 0.83. The Akaike Information Criteria of our model was better than the original four-factor model of Brockington. The present study indicates that the PBQ is a reliable and valid measure of bonding difficulties of Japanese mothers with neonates.

## 1. Introduction

For the last three decades, disorders of the mother–infant relationship, including emotional rejection, have increasingly attracted the attention of clinicians and researchers in perinatal mental health [1,2,3]. A variety of terms have been used to signify these conditions: bonding disorder, mother–infant relationship disorder, and maternal (parental) rejection. The essence of this syndrome is aversion to the infant with marked impairment of interaction [2]. Bonding disorder in mothers after childbirth has been reported as linked to depression [4,5,6,7,8,9,10,11]. However, a recent longitudinal study indicated that bonding disorder and depression shared substantial covariance at one time but are not causal with each other [8,12].

Several self-report instruments have been developed to measure bonding disorder. They include the Maternal Postpartum Attachment Scale [13], the Mother-to-Infant Bonding Scale (MIBS) [14], and the Postpartum Bonding Questionnaire (PBQ) [15]. Scores of these three instruments are moderately correlated [16]. Among them, the PBQ is one of the most extensively studied with regard to its psychometric properties [17,18,19]. Because the PBQ is a self-report questionnaire and is easy to administer, it may be used by obstetric and primary care health professionals as a screening instrument to detect mothers at risk of bonding disorders and child abuse [16].

The validity and reliability of the original English version of the PBQ have been reported by the original author [18]. Brockington and colleagues administered the instrument to 104 mothers and performed a principal component analysis (PCA) of the 84 PBQ items using varimax rotation, this was an orthogonal solution [15]. Through this procedure, they selected 25 items with four main factors (components), including impaired bonding, rejection and anger, anxiety about care, and risk of abuse. When the German version of the PBQ was subjected to a PCA, the original four-factor (component) structure was not confirmed [20]. Unlike exploratory factor analysis (EFA), PCA extracts the first component that explains as much variance of the scale as possible. Hence, the first component is likely to cover many more items than the subsequent components. In order to identify discrete facets of the items of an instrument, EFA is preferable to PCA. To the best of our knowledge, only two studies performed an EFA on the PBQ items. Kaneko and Honjo, using the Kaiser criterion as a measure to define the number of factors, identified five factors with eigenvalues greater than unity [21]. Because the first factor showed a markedly high eigenvalue, they constructed a shorter version with 16 PBQ items that had a single-factor structure. In the other study using the Japanese version of the PBQ [22], an EFA yielded four factors with a shorter version consisting of 14 items. Because these studies concluded different solutions, questions remained as to the factor structure of the Japanese version of the PBQ.

The purpose of this paper is to describe psychometric properties of the Japanese version of the PBQ in terms of its factor structure, construct validity, and test–retest reliability in a community (psychiatrically non-referred) population of Japanese mothers of 1 month-old infants.

## 2. Methods

### 2.1. Participants and Procedure

Of the 55 obstetric clinics in Kumamoto Prefecture, 18 (33%) responded to our request to cooperate with this questionnaire survey. These clinics included one university hospital, twelve public and private hospitals, and five private clinics. Hence, this was a mixture of different types of antenatal institutions in this area. We then solicited the participation of all pregnant women of at least 28 weeks’ gestation who visited one of these obstetrical clinics during the whole month of November 2011. A set of questionnaires was distributed to these women during late pregnancy and again at 5 days (while in the hospital) and 1 month (while attending the one-month health check-up) postnatally. Participating women were asked to complete the questionnaire at home and to return it to the researcher using a stamp-added envelope.

We were interested in the factor structure of the PBQ at 1 month rather than at day 5 after childbirth. This is because the mothers at day 5 are usually still in a secure hospital environment that we hypothesized would mask or eliminate any negative attitudes and emotions towards the baby. Of 1442 eligible women, 392 (27%) returned the questionnaire 1 month after childbirth. These women were the target population of the main analysis in this study. There were 173 first-time mothers, and 180 multipara. The remaining 39 women’s parity was unknown. The mean (SD) number of children they already had had was 0.7 (0.9). Their mean (SD) age was 30.3 (4.9) years. Of them, 98.7% were married. Their partner’s mean age (SD) was 32.2 (6.0) years. In summary, these are not different from mothers in general in Japan [23]. Of these 392 women, 254 (65%) returned the questionnaire 5 days after childbirth, and these were used for test–retest reliability.

### 2.2. Measures

#### 2.2.1. Bonding Disorder

The PBQ was translated into Japanese by Kaneko with the permission of the original author [24]. We used this Japanese version of the PBQ. The PBQ is a self-report instrument that assesses parents’ attitudes and emotions towards their newborn infant. It consists of 25 items rated on a six-point scale (0 to 5). Eight items are positively worded, and these are reverse-scored. Higher scores indicate that the parent has negative affection towards the baby and feels a greater psychological burden with regard to parenting. In this study, the PBQ was distributed to participants at day 5 and at 1 month after childbirth.

#### 2.2.2. Depression

As a measure of depression, we used the Edinburgh Postnatal Depression Scale (EPDS) [25]. The EPDS is a ten item questionnaire rated on a four-point scale (0 to 3), used to assess postnatal depression. Psychometric properties of the original English version of the EPDS have proved to be excellent [25]. Higher scores indicate greater severity of depressive symptoms. The Japanese version of the EPDS is available, and the reliability and validity have been verified [26]. This version has been used in many previous studies by researchers, as well as by maternal health service providers and clinical professionals in community settings in Japan.

#### 2.2.3. Neonatal Abuse

As a measure of neonatal abuse, we used the Conflict Tactics Scale (CTS) [27]. The CTS is a self-report questionnaire that measures the frequency of various abusive parenting behaviors that have occurred since the most recent childbirth. The CTS Child Form R (Parent-child CTS: PCCTS) focuses specifically on parental psychological and physical aggression towards the child. It consists of 19 items rated on a seven-point scale (0: “never”, to 6: “more than 20 times”). The first three items (e.g., “discussed an issue calmly”) comprise the negotiation scale. The others include seven psychological abuse items and nine physical abuse items. In this study, the time frame of the PCCTS was changed from the original of “last year” to “the time period since childbirth”. The PCCTS was translated by one of us (T.K.) after obtaining permission from the original author.

### 2.3. Statistical Analyses

Out of 392 women, 364 (93%) had no missing data in the PBQ and were therefore selected for the present analyses. After randomly dividing these participants into two groups, we examined the means and SDs of all the PBQ items in the first group of mothers (*n* = 172). Because all the PBQ item scores were positively skewed, we conducted a log transformation to achieve an approximate normality assumption. We then performed a series of EFAs on the PBQ items. Because all extracted factors were considered to be interdependent, the factor solution was sought after PROMAX rotation, which is a diagonal rotation method. The number of factors was determined by the Scree test as well as interpretability of the factor structure [28,29].

In order to confirm the stability of the factor structures obtained from the above EFAs, we performed a series of confirmatory factor analyses (CFAs) using another randomly generated subset of participants (*n* = 192). This allowed for cross-validation of the factor structure extracted in the EFA.

We then compared the goodness-of-fit of four models: (a) the model derived from our EFA using the first halved sample; (b) the four-factor model proposed by Brockington et al. [17,18]; (c) the single-factor model proposed by Kaneko and Honjo [21]; and (d) the four-factor model proposed by Suetsugu et al. [22]. We examined the fit of each model with the data in terms of chi-squared (CMIN), root mean square error of approximation (RMSEA), and comparative fit index (CFI). In accordance with conventional criteria, a good fit would be indicated by CMIN/*df* < 2, CFI > 0.97, and RMSEA < 0.05, and an acceptable fit by CMIN/*df* < 3, CFI > 0.95, and RMSEA < 0.08 [30]. We used the Akaike Information Criterion (AIC) [31] as a means of comparing models in terms of goodness of fit. A model is considered superior if its AIC score is lower than that of another model [32].

Internal consistency of the subscales of the PBQ based on the final factor structure was calculated by Cronbach’s alphas.

Construct validity was examined by correlating the scores of the PBQ subscales with the scores of the EPDS and the CTS, measured at 1 month postnatally. This is because we posited that mothers high in bonding difficulty would be more likely to develop depression and more prone to commit neonatal abuse.

Test–retest reliability of the instrument was examined by intraclass correlation coefficient (ICC) between the PBQ subscale scores at 1 month and 5 days after childbirth.

All statistical analyses were conducted using SPSS version 20.0 (IBM Japan, Tokyo, Japan) and Amos 20.0 (IBM Japan).

### 2.4. Ethical Considerations

The study was approved by the Ethical Committee of Kumamoto University Graduate School of Life Sciences, as well as the Institutional Review Board of each institution participating in the study.

## 3. Results

### 3.1. Characteristics of the PBQ Items

Most of the PBQ item scores were low, and 15 of them had a skew of 2.0 or more. However, log transformation of PBQ items resulted in a reduction of skewness (Table 1).

### 3.2. Factor Structure of the PBQ

An EFA of the PBQ items at 1 month after childbirth yielded a three-factor structure (Table 1). The first factor loaded highly (>0.3) on 11 items. An additional two items, though barely reaching a factor loading of 0.3, loaded most highly on the first factor. These 13 items together included those related to mothers’ annoyance with or anger towards their baby, such as “My baby winds me up”, “I feel angry with my baby”, and “My baby irritates me”, as well as items related to mothers feeling that they were “trapped” by parenting—for instance, “I feel trapped as a mother”, “I wish the old days when I had no baby would come back”, and “My baby cries too much”. We named this factor “Anger and Restrictedness”. The second factor loaded highly (>0.3) on six items, including “I love my baby to bits” (reverse item), “I feel happy when my baby smiles or laughs” (reverse item), and “I enjoy playing with my baby” (reverse item). This factor was primarily associated with mothers’ lack of maternal affection and intimacy towards their baby. We named this factor “Lack of Affection”. The third factor loaded highly (>0.3) on five items. They included items such as “I regret having this baby”, and “My baby annoys me”. The other additional item (“The baby does not seem to be mine”) barely reached a loading level of 0.3, but it loaded most highly on the third factor. The third factor appeared to be related to maternal rejection of babies and internal fear. We named this factor “Rejection and Fear”. Hence, all the PBQ items were grouped into one of the three categories. Because bonding disorder is a scarce phenomenon and the PBQ items were considered as its sensitive indicators, we retained all the PBQ items in the EFA.

As a cross validation of the factor structure extracted from the above EFA, we conducted a series of CFA using another randomly generated subset of participants (*n* = 192). This yielded CMIN/*df* = 2.88, CFI = 0.74, and RMSEA = 0.10.

**Figure 1:**
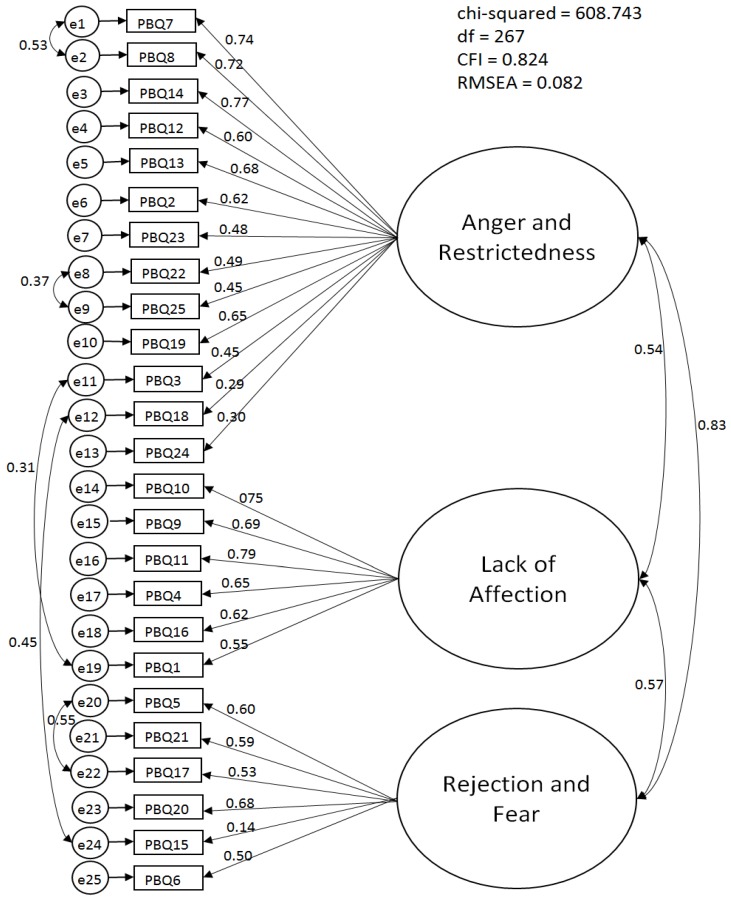
Confirmatory factor analysis of the Postnatal Bonding Questionnaire (PBQ).

Modification indices suggested covariances between some of the error variables. Adding these covariances, we obtained a final model with better indices of fit: CMIN/*df* = 2.28, CFI = 0.82, and RMSEA = 0.08 (Figure 1). All the coefficients of paths from the latent variable were statistically significant, except for item 15 (standardized path coefficient = 0.14). The three latent variables were significantly correlated with each other: Anger and Restrictedness and Lack of Affection, *r* = 0.54; Anger and Restrictedness and Rejection and Fear, *r* = 0.83; and Lack of Affection and Rejection and Fear, *r* = 0.57.

In order to compare the four models (i.e., (a) the model derived from our EFA using the first halved sample; (b) the four-factor model proposed by Brockington et al. [17,18]; (c) the single-factor model proposed by Kaneko and Honjo [21]; and (d) the four-factor model proposed by Suetsugu et al. [22]), we conducted a CFA of each model using the second half-sample (Table 2). None of them reached the acceptable level of goodness of fit. However, the AIC showed that the four-factor model proposed by Suetsugu et al. was the best. Nevertheless, the model by Suetsugu et al. trimmed the number of the PBQ items to 14. Kaneko and Honjo also reduced the number of items to 16. Using all the PBQ items, our EFA-derived model showed a better fit than Brockngton et al.’s original model. Therefore, we concluded that our three-factor model has the possibility to describe the present data best. Subsequent analyses were conducted using the three-factor model.

Cronbach’s alpha coefficients of Anger and Restrictedness, Lack of Affection, and Rejection and Fear were 0.81, 0.82, and 0.64, respectively, in our three-factor model.

### 3.3. Test–Retest Reliability

Test–retest reliabilities of the three PBQ subscales were substantial among the 254 women who completed the PBQ at both 1 month and 5 days after childbirth: Anger and Restrictedness, ICC = 0.83; Lack of Affection, ICC = 0.82; and Rejection and Fear, ICC = 0.76 (Table 3).

### 3.4. Construct Validity

Among our participants, 23 mothers (5.2%) scored above 10 on the EPDS, while six mothers (0.8%) scored 13 or greater. The EPDS score was significantly correlated with each of the PBQ subscale scores at 1 month after childbirth (Table 4). The scores of the psychological abuse scale of the CTS were significantly correlated with each of the PBQ subscales. On the other hand, the scores of the physical abuse scale of the CTS were not correlated with any of the PBQ subscales.

## 4. Discussion

The present study demonstrated that there were three domains of PBQ items, each corresponding to different aspects of mothers’ attitudes and emotions towards their babies: Anger and Restrictedness, Lack of Affection, and Rejection and Fear. When compared with the four models presented previously, we believe that our model showed reasonable fit with the data.

There may be several possible reasons why the factor structure we identified differed from those in prior studies. First, Brockington et al. used a patient population, while our study used a community population [15]. This may be associated with the fact that the PBQ items were positively skewed in our study. Second, as noted earlier, Brockington et al. used a PCA as a means to categorize the PBQ items. This is a technique that defines the first component in such a way that it has the largest possible variance. Thus, the first component captures much of the information originally contained in the items of the instrument [33]. A PCA is more likely to produce a general rather than specific factor. Thus, four PBQ items that loaded most highly on the first factor (“impaired bonding”) in Brockington et al.’s report were scattered into three different factors in our study. Thus the item “I feel happy when my baby smiles or laughs” loaded on the second factor, the items “the baby does not seem to be mine” and “I wish my baby would somehow go away” loaded on the third factor, and the item “my baby winds me up” loaded on the first factor.

Kaneko and Honjo [21], Suetsugu et al. [22], and our group all used a non-clinical Japanese mother population. Kaneko and Honjo suggested a single-factor structure because the first factor showed a markedly high eigenvalue. They then produced a shorter version of the PBQ by selecting only items with high factor loadings on the first factor in their study. The strength of their study is the large sample size (*n* = 1786). In contrast, the study of Suetsugu et al., based on a relatively small sample (*n* = 244), suggested a four-factor structure. This was derived through deletion of some items that were considered to be non-adaptable because of low factor loading values (≤0.35). Although deletion of items may, by definition, result in better internal consistency, such procedures are not necessarily free from flaws. Psychological phenomena such as bonding disorder are likely to have different facets that may yield different factors obtained through EFAs. Reducing the number of items in an instrument may sacrifice such diversity. Hence, the full version of the PBQ should be retained in order to identify multiple aspects of bonding disorder.

Another important research issue is cross-validation of the factor structure. The factor structure derived from an EFA should be validated by means of a CFA of a sample different from the one that yielded the EFA. Neither Kaneko and Honjo [21] nor Suetsugu et al. [22] performed CFAs. A strength of our study is a CFA suggesting a reasonably robust factor structure of the PBQ. Because our Three-factor model contained the full 25 items and utilized factor analysis rather than a PCA together with cross-validation via a CFA, it may be superior to the original model of Brockington et al. [15,18] in our population of Japanese mothers of neonates.

While test–retest reliability of the three PBQ subscales was substantial, the scores of Anger and Restrictedness increased from day 5 to 1 month. In Japan, most mothers are still in a hospital at 5 days after childbirth. Hence, they receive ample support and do not face the burdens of household chores. This is in contrast to the situation 1 month after childbirth, when restricted and angry feelings towards the baby are more likely to surface. On the other hand, Lack of Affection and Rejection and Fear may reflect a more psychological refusal unrelated to the living environment. Hence clinicians should pay more attention to Lack of Affection and Rejection and Fear as a means of identifying mothers who need psychological support in the early stage after delivery.

As expected from previous investigations [34,35,36], all three PBQ subscale scores were associated with EPDS and CTS psychological abuse scores. Among the PBQ subscales, Anger and Restrictedness was markedly related to the EPDS total scores and the psychological abuse scores of the CTS. This result supports a previous report that EPDS was strongly associated with anger and rejection in the Mother-to-Infant Bonding Scale (MIBS) at 1 month after childbirth [35]. Lack of association between the physical abuse subscale of the CTS and the PBQ may be due to a narrower variation (SD = 0.05) of the physical abuse scores in our sample. We think that our three-factor PBQ model has good construct validity.

The three subscales of the PBQ were clinically interpretable. Anger and Restrictedness may be linked to maternal burdens from and restriction in child rearing, resulting in various stress-related psychopathologies. For example, maternal parenting stress may be related to trait anger and to maternal emotional stress and negative affection towards the baby. The second subscale, Lack of Affection, consists of reverse items referring to affection for the baby.

Rejection and Fear of mothers may reflect the “ghost in the nursery” [37]. This term refers to the clinical observation that the presence of a baby may sometimes arouse unconscious negative feelings in the mother, such as fear, agitation, and anxiety. This stems from their past painful experience and repressed affections that are easily projected to their infants [37,38,39].

Despite the difference of the three subscales, these were moderately correlated. This suggests a generic concept of bonding failure itself.

A drawback of our study is that a CFA showed that our model fit only moderately well with the current data, which limits generalization. This may be due to our relatively small sample size and the use of a community population. Further, our study had a low response rate, which might have impeded our identification of the target population of women with bonding disorder. Our data may therefore be subjected to selection bias. Brockington [2] claimed that a population of more than 1000 would be needed for this type of study, due to the infrequency of bonding disorders. Because of our use of a nonclinical population, the data were very positively skewed. The log transformation of the data improved normality, but positive skew remained for some items. Mothers whose child is under temporary custody at Child Protection Centre may provide more normal distribution of the PBQ scores. Such a population is to be studied in future studies.

## 5. Conclusions

Taking the above drawbacks into consideration, the present study indicates that the PBQ is a promising measure of bonding difficulties in Japanese mothers with neonates. Considering the importance of maternal bonding towards their infant in health care and health policy making, we believe that the development and standardization of instruments, including the PBQ, will promote further clinical endeavors to treat and prevent postnatal bonding disorder.

## Figures and Tables

**Table 1:** Exploratory factor analysis of the PBQ items in a split-half sample (*n =* 172).

Item Number	PBQ Items	Mean (SD)	Skewness	Skewness after Log Transformation	Commu-nality	Factor		
						I	II	III
7	My baby winds me up	0.81 (0.81)	0.4	0.43	0.64	0.83	−0.14	0.01
8	My baby irritates me	0.88 (0.82)	0.2	0.2	0.56	0.78	−0.09	−0.02
14	I feel angry with my baby	0.32 (0.54)	1.5	1.5	0.58	0.70	−0.08	0.16
12	My baby cries too much	1.15 (1.13)	1.0	0.3	0.30	0.58	0.07	−0.21
13	I feel trapped as a mother	1.08 (1.20)	1.3	0.3	0.31	0.55	0.02	0.02
2	I wish the old days when I had no baby would come back	0.55 (0.73)	0.9	1.1	0.36	0.48	0.14	0.10
23	I feel the only solution is for someone else to look after my baby	0.85 (0.96)	1.1	0.5	0.22	0.45	0.01	0.04
22	I feel confident when changing my baby	2.14 (1.44)	0.1	−0.7	0.21	0.44	0.10	−0.06
25	My baby is easily comforted	1.99 (1.22)	0.2	−0.5	0.22	0.44	0.14	−0.07
19	My baby makes me anxious	0.40 (0.70)	1.6	1.3	0.20	0.37	0.10	0.06
3	I feel distant from my baby	0.45 (0.99)	2.9	1.7	0.19	0.31	0.15	0.09
18	I have done harmful things to my baby	0.03 (0.17)	5.7	7.9	0.12	0.27	−0.10	0.16
24	I feel like hurting my baby	0.01 (0.08)	13.1	6.0	0.03	0.20	0.00	−0.08
10	I love my baby to bits	0.32 (0.78)	3.5	2.0	0.78	−0.02	0.92	−0.09
9	I feel happy when my baby smiles or laughs	0.15 (0.66)	5.8	4.8	0.57	−0.23	0.79	0.01
11	I enjoy playing with my baby	0.40 (0.81)	2.6	1.8	0.67	0.18	0.76	−0.03
4	I love to cuddle my baby	0.27 (0.80)	4.3	2.5	0.33	0.04	0.54	0.04
16	My baby is the most beautiful baby in the world	0.37 (0.85)	2.8	1.9	0.33	0.11	0.51	0.06
1	I feel close to my baby	0.28 (0.61)	2.5	1.86	0.43	0.18	0.47	0.18
5	I regret having this baby	0.03 (0.21)	6.8	4.70	0.75	−0.10	−0.02	0.91
21	My baby annoys me	0.08 (0.33)	4.4	3.36	0.68	0.00	−0.06	0.84
17	I wish my baby would somehow go away	0.02 (0.13)	7.4	4.93	0.58	−0.14	0.13	0.76
20	I am afraid of my baby	0.05 (0.28)	7.9	3.66	0.32	−0.02	0.01	0.58
15	I resent my baby	0.05 (0.21)	4.4	8.25	0.26	0.17	−0.05	0.42
6	The baby does not seem to be mine	0.17 (0.67)	5.4	2.22	0.14	0.08	0.12	0.26

**Table 2:** Comparison of three models of the PBQ factor structure.

Goodness of Fit Indices	Three-Factor Model Derived from the EFA in This Study	Four-Factor Model by Brockington et al.	One-Factor Model by Kaneko and Honjo	Four-Factor Model by Suetsugu et al.
CMIN	608.74	1043.20	409.99	172.44
*Df*	267	270	104	71
CMIN/*df*	2.28	3.86	3.94	2.43
CFI	0.82	0.60	0.73	0.90
RMSEA	0.08	0.12	0.12	0.09
AIC	724.74	1153.2	473.99	240.44

CMIN, chi-squared; CFI, comparative fit index; RMSEA, root mean square error of approximation; AIC, Akaike information criteria. EFA: exploratory factor analysis.

**Table 3:** Test–retest reliability of the PBQ factors (Intraclass Correlation Coefficient; ICC).

PBQ Subscales	1 Month After Childbirth	5 Days After Childbirth	ICC	*F*
	Mean (SD)	Mean (SD)		
Anger and Restrictedness	10.24 (6.92)	9.13 (6.46)	0.83	6.11 ***
Lack of Affection	1.69 (3.37)	1.85 (3.52)	0.82	5.69 ***
Rejection and Fear	0.49 (1.41)	0.49 (1.35)	0.76	4.21 ***

*** *p* < 0.001.

**Table 4:** Correlations between each factor of the PBQ and other scale scores.

Instruments	Mean (SD)	PBQ Subscales		
		Anger and Restrictedness	Lack of Affection	Rejection and Fear
EPDS	3.07 (3.28)	0.49 **	0.21 **	0.32 **
Psychological abuse	8.01 (2.13)	0.45 **	0.29 **	0.31 **
Physical abuse	9.00 (0.05)	0.10	0.01	−0.01

EPDS: Edinburgh Postnatal Depression Scale. ** *p* < 0.01.

## References

[1] Brockington I.F. (2004). Diagnosis and management of post-partum disorders: A review. World Psychiatr..

[2] Brockington I.F. (2011). Maternal rejection of the young child: Present status of the clinical syndrome. Psychopathology.

[3] Kumar R.C. (1997). “Anybody’s child”: Severe disorders of mother-to-infant bonding. Br. J. Psychiatr..

[4] Edhborg M., Matthiesen A.S., Lundh W., Widström A.M. (2005). Some early indicators for depressive symptoms and bonding 2 months postpartum: A study of new mothers and fathers. Arch. Womens Ment. Health.

[5] Edhborg M., Nasreen H.E., Kabir Z.N. (2011). Impact of postpartum depressive and anxiety symptoms on mothers’ emotional tie to their infants 2–3 months postpartum: A population-based study from rural Bangladesh. Arch. Womens Ment. Health.

[6] Honjo S., Arai S., Kaneko H., Ujiie T., Murase S., Sechiyama H., Inoko K. (2003). Antenatal depression and maternal-fetal attachment. Psychopathology.

[7] Klier C.M. (2006). Mother-infant bonding disorders in patients with postnatal depression: The Postpartum Bonding Questionnaire in clinical practice. Arch. Womens Ment. Health.

[8] Kokubu M., Okano T., Sugiyama T. (2012). Postnatal depression, maternal bonding failure, and negative attitudes towards pregnancy: A longitudinal study of pregnant women in Japan. Arch. Womens Ment. Health.

[9] Moehler E., Brunner R., Wiebel A., Reck C., Resch F. (2006). Maternal depressive symptoms in the postnatal period are associated with long-term impairment of mother-child bonding. Arch. Womens Ment. Health.

[10] Nagata M., Nagai Y., Sobajima H., Ando T., Honjo S. (2003). Depression in the mother and maternal attachment: Results from a follow-up study at 1 year postpartum. Psychopathology.

[11] Nagata M., Nagai Y., Sobajima H., Ando T., Nishide Y., Honjo S. (2000). Maternity blues and attachment to children in mothers of full-term normal infants. Acta Psychiatr. Scand..

[12] Ohashi Y., Kitamura T., Sakanashi K., Tanaka T. Jido gyakutai no genin wa sango no yokuutsu de wa naku bondingu shougai de aru: Kumamoto chiku no jyudan chousa kara (A cause of the neonatal abuse was the bonding disorders not postpartum depression: A longitudinal study in Kumamoto prefecture). Proceedings of the Poster Session on the 11th Academic Meeting of the Japanese Society of Perinatal Mental Health.

[13] Condon J.T., Corkindale C. (1998). The assessment of parent-to-infant attachment: Development of a self-report questionnaire measurement. J. Reprod. Infant Psychol..

[14] Tayler A., Atkins R., Kumar R., Adams D., Glover V. (2005). A new mother-to-infant bonding scale: Links with early maternal mood. Arch. Womens Ment. Health.

[15] Brockington I.F., Oats J., George S., Turner D., Vostanis P., Sullivan M., Murdoch C. (2001). A screening questionnaire for mother-infant bonding disorders. Arch. Womens Ment. Health.

[16] Van Bussel J.C.H., Spitz B., Demyttenaere K. (2010). Three self-report questionnaires of the early mother-to-infant bond: Reliability and validity of the Dutch version of the MPAS, PBQ and MIBS. Arch. Womens Ment. Health.

[17] Brockington I.F., Aucamp H.M., Fraser C. (2006). Severe disorders of the mother-infant relationship: Definition and frequency. Arch. Womens Ment. Health.

[18] Brockington I.F., Fraser C., Wilson D. (2006). The postpartum bonding questionnaire: A validation. Arch. Womens Ment. Health.

[19] Wittkowski A., Wiek A., Mann S. (2007). An evaluation of two bonding questionnaires: A comparison of the mother-to-infant bonding scale with the postpartum bonding questionnaire in a sample of primiparous mothers. Arch. Womens Ment. Health.

[20] Reck C., Klier C.M., Pabst K., Stehle E., Steffenelli U., Struben K., Backenstrass M. (2006). The German version of the postpartum Bonding Instrument: Psychometric properties and association with postpartum depression. Arch. Womens Ment. Health.

[21] Kaneko H., Honjo S. (2014). The psychometric properties and factor structure of the Postpartum Bonding Questionnaire in Japanese mothers. Psychology.

[22] Suetsugu Y., Honjo S., Ikeda M., Kamibeppu K. (2015). The Japanese version of the Postpartum Bonding Questionnaire: Examination of the reliability, validity, and scale structure. J. Psychosom. Res..

[23] Ministry of Health, Labour and Welfare Annual Health, Labour and Welfare Report 2012–2013 (Summary), 2013. http://www.mhlw.go.jp/english/wp/wp-hw7/dl/summary.pdf.

[24] Kaneko H. Early Intervention and Support System for Postpartum Depression and Postpartum Bonding Disorders.

[25] Cox J.L., Holden M., Sagovsky R. (1987). Detection of postnatal depression development of the 10-item Edinburgh Postnatal Depression Scale. Br. J. Psychiatr..

[26] Okano T., Masuji F., Tamaki R., Nomura J., Murata M., Miyaoka H., Kitamura T. (1996). Validation and reliability of Japanese version of the Edinburgh Postnatal Depression Scale (EPDS). Arch. Psychiatr. Diagnos. Clin. Eval..

[27] Straus M.A., Hamby S.L. (1995). Measuring Physical and Psychological Maltreatment of Children with the Conflict Tactics Scales. Manual for the Conflict Tactics Scales (CTS) and Test Forms for the Revised Conflict Tactics Scales.

[28] Cattell R.B. (1966). The Scree test for the number of factors. Multivar. Behav. Res..

[29] Zwick W.R., Velicer W.F. (1982). Factors influencing four rules for determining the number of components to retain. Multivar. Behav. Res..

[30] Schermelleh-Engell K., Moosbrugger H., Müller H. (2003). Evaluating the fit of structural equation models: Tests of significance and descriptive goodness-of-fit measures. Method. Psychol. Res. Online.

[31] Akaike H. (1987). Factor analysis and AIC. Psychometrika.

[32] Kline R.B. (2005). Principles and Practice of Structural Equation Modelling.

[33] DeVellis R.F. (2012). Scale Development: Theory and Applications.

[34] Kitamura T., Ohashi Y., Kita S., Haruna M., Kubo R. (2013). Depressive mood, bonding failure, and abusive parenting among mothers with three-month-old babies in a Japanese community. Open J. Psychiatr..

[35] O’Higgins M., Roberts I.S.J., Glover V., Taylor A. (2013). Mother-child bonding at 1 year: Associations with symptoms of postnatal depression and bonding in the first few weeks. Arch. Womens Ment. Health.

[36] Yoshida K., Yamashita H., Conroy S.A. (2012). Japanese version of Mother-to-Infant Bonding Scale: Factor structure, longitudinal changes and links with maternal mood during the early postnatal period in Japanese mothers. Arch. Womens Ment. Health.

[37] Fraiberg S., Adelson E., Shapiro V. (1975). Ghosts in the nursery: A psychoanalytic approach to problems of impaired infant-mother relationships. J. Am. Acad. Child Adolesc. Psychiatr..

[38] Watanabe H., Watanabe H., Hashimoto Y. (2001). Nyu-yo-ji seisin hoken no atarasii doukou (The new trend of infant mental health). Nyu-yo-ji Seisin Hoken No Atarasii Kaze (The New Tide of Infant Mental Health).

[39] Slade A., Cohen L.J., Sadler L.J., Miller M., Zeanah C.H. (2009). The psychology and psychopathology of pregnancy. Handbook of Infant Mental Health.

